# Epidemiology and Antibiotic Resistance Patterns of Klebsiella pneumoniae Infections Among Male Patients in a Long-Term Care Hospital in Riyadh, Saudi Arabia: A Retrospective Study

**DOI:** 10.7759/cureus.72101

**Published:** 2024-10-22

**Authors:** Badriah A Alfaifi, Saleh A Alkhaldi, Majed D Alanazi, Wadi A Shuraim, Masoud A Aldossry, Hanan S Alzahrani, Fatoom A Alhaitei

**Affiliations:** 1 Infection Control, Long Term Care Hospital, Riyadh, SAU; 2 Research Center, King Saud Medical City, Riyadh, SAU; 3 Internal Medicine, Long Term Care Hospital, Riyadh, SAU

**Keywords:** antibiotic resistance, carbapenem-resistant enterobacterales, extended-spectrum beta-lactamases (esbl), klebsiella pneumoniae, long-term care, saudi arabia

## Abstract

Background: *Klebsiella pneumoniae* is an opportunistic pathogen commonly found in the normal flora of the gastrointestinal tract. While it typically remains benign, it can lead to severe healthcare-associated infections, particularly among immunocompromised individuals and those in long-term care settings. This study aimed to investigate the epidemiology and antibiotic resistance patterns of *K. pneumoniae* infections among male patients in a long-term care hospital.

Methods: A retrospective cross-sectional study was conducted, analyzing microbial culture data from 29 male patients admitted to the male ward of a long-term care hospital in Riyadh, Saudi Arabia. The study included patients with confirmed positive cultures for *K. pneumoniae*. Data were collected regarding demographic information, culture sites, and antibiotic susceptibility test results. Statistical analysis was performed to identify significant associations between patient characteristics and resistance patterns, with a particular focus on the prevalence of carbapenem-resistant Enterobacterales (CRE) and the production of extended-spectrum beta-lactamases (ESBL).

Results: Around 51.7% of the *K. pneumoniae* isolates were found to be resistant to carbapenems, while 24.1% were classified as producers of ESBL. Patients suffering from bedsores had a significantly higher prevalence of CRE infections at 37.9% compared to only 13.8% in patients without bedsores (p=0.034). The analysis also indicated that older patients (aged over 50 years) exhibited a higher prevalence of CRE infections (34.5%) compared to their younger counterparts (17.2%, p=0.069). Notably, resistance levels against antibiotics were high, with 75.9% of isolates resistant to cotrimoxazole, 65.5% to ciprofloxacin, and 62.1% to gentamicin. In contrast, imipenem and meropenem showed relatively higher susceptibility, with each antibiotic having a susceptibility rate of 34.5%.

Conclusion: There is a significant prevalence of multidrug-resistant *K. pneumoniae *infections in long-term care environments, particularly affecting older male patients and those with bedsores. These findings underscore the need for targeted infection control measures and enhanced antibiotic stewardship programs. Future research should include both genders to investigate epidemiological differences and broader resistance trends.

## Introduction

*Klebsiella pneumoniae* is a part of the normal flora that typically inhabits the human gastrointestinal and oropharyngeal tracts [1,2]. However, under certain conditions, it can shift from being harmless to causing severe infections in other tissues [3]. It significantly contributes to healthcare-associated infections, particularly due to its growing resistance to antibiotics. This bacterium, classified as gram-negative, is one of the most common pathogens responsible for bloodstream infections (BSIs), trailing only *Escherichia coli*. BSIs can manifest as primary infections without an identifiable source but are often linked to the spread of pathogens from known infection sites such as the urinary tract, gastrointestinal system, intravenous or urinary catheters, and respiratory sites [4,5].

Various underlying conditions increase the risk of *K. pneumoniae* infections. These include diabetes mellitus, liver diseases, and cancerous tumors, all of which account for around 50% of community-acquired BSIs caused by *K. pneumoniae* [6,7]. A previous study also identified chronic lung disease as a significant contributing factor, especially in elderly patients. Other contributing factors include prior antibiotic use and invasive medical procedures, such as endotracheal intubation, bladder catheterization, and intravenous catheterization, which substantially elevate infection risk [8]. Additionally, hypervirulent strains of *K. pneumoniae* are known to cause severe disseminated infections, including liver and prostate abscesses, pneumonia, meningitis, and endophthalmitis [9].

Over the past two decades, *K. pneumoniae* has garnered increasing attention due to its escalating levels of antibiotic resistance and the serious health complications it causes [9,10]. This pathogen is now one of the leading causes of hospital-acquired pneumonia, responsible for around 10% of all hospital-acquired infections, and is the second most common gram-negative bacterium in such settings [11,12]. Multidrug-resistant (MDR) *K. pneumonia* strains are particularly problematic, contributing to various healthcare-associated infections such as pneumonia, urinary tract infections (UTIs), and BSIs [13]. These MDR *K. pneumoniae* strains produce extended-spectrum beta-lactamases (ESBL) and carbapenemases, leading to increased mortality rates, particularly among immunocompromised and critically ill patients [14]. Moreover, the emergence of extensively drug-resistant (XDR) and pan-drug-resistant strains presents an even greater challenge, as these strains demonstrate resistance to nearly all available antimicrobial agents [9,15].

Furthermore, epidemiological evidence suggests a global spread of hypervirulent and MDR *K. pneumoniae* strains, particularly in Southeast Asia, with significant clinical implications. These strains are associated with severe infections, including pneumonia, UTIs, and BSIs, which are increasingly resistant to last-resort antibiotics such as carbapenems [16]. This alarming trend underscores the need for robust infection control measures and antimicrobial stewardship programs, particularly in long-term care facilities, where patients are at heightened risk [16].

Long-term care facilities house a high concentration of frail patients with chronic medical conditions, placing them at significant risk for infections [17]. A longitudinal study observed that patients colonized with *K. pneumoniae* were more likely to develop respiratory system infections, UTIs, or BSIs. Specifically, 5.2% of colonized patients developed subsequent infections, compared to only 1.3% of non-colonized patients. This data highlights the importance of monitoring colonization in high-risk healthcare settings [18,19].

The rising prevalence of MDR *K. pneumoniae* infections has prompted the World Health Organization (WHO) to classify this pathogen as a critical priority, given its public health implications [20]. Studies in the country have mirrored these global concerns, with MDR *K. pneumoniae* strains becoming increasingly prevalent in healthcare settings [21,22].

We investigated the epidemiological characteristics and antibiotic resistance patterns of *K. pneumoniae *infections among male patients in a long-term care hospital in Riyadh, Saudi Arabia. The study focused on the prevalence of infections, associated risk factors, antimicrobial resistance profiles, and treatment outcomes. The findings aim to inform targeted interventions and policies to reduce the spread of *K. pneumoniae* and improve patient care in this facility. 

Study objectives

(i) To analyze the epidemiological evolution of *K. pneumoniae* infections in a male ward at Riyadh's Long Term Care Hospital, Saudi Arabia. (ii) To determine the antibiotic resistance patterns of MDR *K. pneumoniae* infections in male subjects hospitalized in the facility.

## Materials and methods

This retrospective cross-sectional study analyzed *K. pneumoniae* microbial culture data from the male ward at Riyadh's Long Term Care Hospital. The study included male patients with confirmed positive *K. pneumoniae* cultures obtained between January 1, 2023, and December 31, 2023. The focus on male patients was due to ongoing maintenance work in the female ward, which prevented the collection of female data during the study period. However, a follow-up study is planned to include female patients. Inclusion criteria included all male patients hospitalized at the facility during the study period with confirmed *K. pneumoniae* infections based on positive microbial culture results. Cultures from outpatients and long-term care patients with non-pathogenic *K. pneumoniae* colonization were excluded.

Data were collected from paper-based microbial culture records maintained by the infection control department. A total of 29 samples from 29 male patients who met the inclusion criteria were included in the study. These data were transcribed into a dedicated Excel spreadsheet (Microsoft Corp., Redmond, USA) for analysis. Data points included patient demographics, culture site, antibiotic susceptibility testing results, and 30-day mortality data related to carbapenem-resistant Enterobacterales (CRE). Due to technical reasons related to the study site at the time of data collection, only male patients were included. However, there is a plan to conduct another study on female patients in the near future.

Antimicrobial susceptibility testing was conducted using the disc diffusion method, according to the guidelines provided by the Clinical and Laboratory Standards Institute (CLSI) [23]. The CLSI guidelines outline standard procedures for determining microbial resistance to antibiotics by measuring the zone of inhibition around the antibiotic discs [23]. The results from this testing were used to determine the resistance patterns of the *K. pneumoniae *strains against several antibiotics, totaling 17 different antibiotics.

The study received ethical approval (#H1RI-17-Mar24-01) from the Institutional Review Board at the Saudi Ministry of Health. It adhered to principles outlined in the Declaration of Helsinki, current legislation on clinical research, and Good Clinical Practice guidelines. All patient data were kept confidential throughout the study.

Descriptive statistics characterized patient demographics, culture site distribution, and antibiotic resistance patterns. Chi-square tests explored potential associations among medical factors, CRE prevalence, and ESBL resistance patterns in *K. pneumoniae* infections. IBM SPSS Statistics for Windows, version 24 (IBM Corp., Armonk, USA) was used to perform statistical analysis. A p-value of less than 0.05 was considered statistically significant.

## Results

A total of 29 samples were obtained from 29 male patients with a mean age of 45.76 years (±15.64). Among them, 48.3% were aged between 20 and 50 years, while 51.7% were over 50 years old. The average hospital stay duration was 861.93 days (±1610.74 days), where 55.2% spent less than 200 days in the hospital, and 44.8% spent more than 200 days. Furthermore, 55.2% had bedsores, 69.0% had a tracheostomy, and all had Foley catheters. Specimen cultures were taken from blood (3.4%), sputum (20.7%), urine (37.9%), and 37.9% wounds. There were no deaths within 30 days of study discontinuation. Around 51.7% of the patients were resistant to CRE, 24.1% to ESBL, and 24.1% had unknown resistances (Table 1).

**Table 1:** Clinical characteristics of patients with Klebsiella pneumoniae (n=29) This table presents a comprehensive overview of the characteristics of male patients with *K. pneumoniae* infections in a long-term care hospital in Riyadh, Saudi Arabia. CRE: Carbapenem resistance; ESBL: Extended-spectrum beta-lactamases

Characteristics	Frequency	Percent
Age		
From 20-50 years	14	48.3%
Over 50 years	15	51.7%
Hospital stay duration		
Under 200 days	16	55.2%
Over 200 days	13	44.8%
Bedsore(s)		
Yes	16	55.2%
No	13	44.8%
Foley catheter		
Yes	29	100%
Tracheostomy		
Yes	20	69%
No	9	31%
Culture site		
Blood	1	3.4%
Sputum	6	20.7%
Urine	11	37.9%
Wound	11	37.9%
Antibiotic resistance		
CRE	15	51.7%
ESBL	7	24.1%
Unknown	7	24.1%
Death within 30 days from CRE		
No	29	100%

Antimicrobial susceptibility analysis of the bacterial isolates revealed a disturbing trend of increasing resistance levels, particularly towards commonly used antibiotics. High levels of resistance were observed for cotrimoxazole (75.9%), ciprofloxacin (65.5%), gentamicin (62.1%), imipenem (55.2%), meropenem (48.3%), and amikacin (24.1%). Other antibiotics with lower resistance levels included cefepime (34.5%), Tazocin (27.6%), and ertapenem (27.6%). Imipenem and meropenem exhibited the highest susceptibility percentage at 34.5% each, making them potential treatment options. Tigecycline also showed no resistance, with 6.9% intermediate susceptibility and 20.7% susceptibility. Other antibiotics like ceftriaxone, cefotaxime, and ampicillin displayed high resistance without intermediate or susceptible isolates (Table 2).

**Table 2:** Antimicrobial susceptibility testing for Klebsiella pneumoniae (n=29) This table provides a detailed breakdown of the antimicrobial susceptibility testing results for the 29 *K. pneumoniae* isolates obtained from the study participants. Antibiotics are classified based on their type, and the percentage (%) of resistance, intermediate susceptibility, and susceptibility are determined for each antibiotic.

Type		Resistant	Intermediate	Sensitive
Ampicillin	Fre (%)	2 (6.9%)	-	-
Augmentin	Fre (%)	2 (6.9%)	-	1 (3.4%)
Amikacin	Fre (%)	14 (48.3%)	-	7 (24.1%)
Cefepime	Fre (%)	10 (34.5%)	-	2 (6.9%)
Cefotaxime	Fre (%)	2 (6.9%)	-	-
Ceflazidme	Fre (%)	6 (20.7%)	-	1 (3.4%)
Cerftriaxone	Fre (%)	4 (13.8%)	-	-
Ciprofloxacin	Fre (%)	19 (65.5%)	-	6 (20.7%)
Cotrimoxazole	Fre (%)	22 (75.9%)	-	7 (24.1%)
Gentamicin	Fre (%)	18 (62.1%)	-	8 (27.6%)
Imipenem	Fre (%)	16 (55.2%)	-	10 (34.5%)
Meropenem	Fre (%)	14 (48.3%)	-	10 (34.5%)
Ertapenem	Fre (%)	8 (27.6%)	1 (3.4)	-
Tazocin	Fre (%)	8 (27.6%)	-	-
Colistin	Fre (%)	1 (3.4%)	4 (13.8)	1 (3.4%)
Tigecycline	Fre (%)	-	2 (6.9)	6 (20.7%)
Nitrofurantoin	Fre (%)	-	8 (27.6)	1 (3.4%)
Total	Fre (%)	146 (29.6%)	15 (3%)	60 (12.17%)

As shown in Table 3, we found a significant association between bedsores and CRE (p=0.034). Patients with bedsores were significantly more likely to demonstrate CRE infections (38%) than those without (13.8%). Conversely, ESBL-producing isolates were more common (20.7) in patients without bedsores (in contrast to 3.44% in patients with bedsores). Thus, bedsores may increase the likelihood of developing CRE infections in this patient population.

**Table 3:** Medical and other factors associated with CRE and ESBL resistance patterns in patients with Klebsiella pneumoniae infections This table demonstrates associations between *K. pneumoniae* infections with CRE prevalence and ESBL resistance patterns and identifies potential risk factors for MDR strains. CRE: Carbapenem resistance; ESBL: Extended-spectrum beta-lactamases; MDR: Multidrug-resistant

Items	Characteristic	CRE	ESBL	Unknown	Total	P-value
Bedsores	Yes	11 (38%)	1 (3.44%)	4 (13.8%)	16 (55.2%)	0.034
	No	4 (13.8%)	6 (20.7%)	3 (10.3%)	13 (44.8%)	
Foley catheter	Yes	15 (51.7%)	7 (24.1%)	7 (24.1%)	29 (100%)	--
Tracheostomy	Yes	12 (41.4%)	5 (17.24%)	3 (10.3%)	20 (69%)	0.212
	No	3 (33.3%)	2 (6.9%)	4 (13.8%)	9 (31%)	
Culture site	Blood	0 (0%)	1 (3.4%)	0 (0%)	1 (3.4%)	0.098
	Sputum	4 (13.8%)	1 (3.4%)	1 (3.4%)	6 (20.7%)	
	Urine	3 (10.3%)	5 (17.24%)	3 (10.3%)	11 (37.9%)	
	Wound	8 (53.3%)	0 (0%)	3 (10.3%)	11 (37.9%)	
Age	Younger (<51 years)	5 (17.24%)	6 (20.7%)	3 (10.3%)	14 (48.3%)	0.069
	Older (>50 years)	10 (34.5%)	1 (3.4%)	4 (13.8%)	15 (51.7%)	
Hospital stay duration	Fewer than 200 days	8 (27.6%)	3 (10.3%)	5 (17.24%)	16 (55.2%)	0.549
	More than 200 days	7 (24.1%)	4 (13.8%)	2 (6.9%)	13 (44.8%)	

A tracheostomy was not significantly associated with CRE or ESBL infections (p=0.212). Patients with a tracheostomy were more likely to demonstrate CRE (41.4%) than those without (33.3%). The distribution of resistance patterns also varied relative to culture site; CRE was more prevalent in wound cultures (53.3%), and ESBL-producing isolates were more common in urine cultures (17.24%). However, the association between culture site and resistance patterns was non-significant (p=0.098).

Patients older than 50 had more CRE (34.5%) than their younger counterparts (17.24%). In contrast, ESBL-producing isolates were more common in younger (20.7%) versus older (3.4%) patients. The association between age and resistance patterns approached statistical significance (p=0.069); thus, better-powered studies may reveal older age as a risk factor for CRE infections.

The hospital stay duration (fewer or more than 200 days) was not significantly associated with the prevalence of CRE or ESBL resistance patterns (p=0.549). Patients with longer hospital stays (more than 200 days) had a slightly higher proportion of ESBL-producing isolates (13.8%) compared to those with shorter stays (10.3%) (Table 3).

Importantly, all subjects had Foley catheters, a well-known risk factor for healthcare-associated infections. Our results do not include a statistical analysis of the association between Foley catheter use and the resistance patterns, as these catheters were ubiquitous (Table 1).

## Discussion

The current study investigated the epidemiological characteristics and antibiotic resistance patterns of *K. pneumoniae *infections among male patients hospitalized in a long-term care hospital in Riyadh, Saudi Arabia. We also aimed to analyze the evolution of epidemiology and determine *K. pneumoniae*’s antibiotic resistance in this setting.

The study’s main findings revealed a high prevalence (>50%) of CRE among the patients. Moreover, 24.1% of patients had ESBL-producing *K. pneumoniae*. This is a concerning trend as CRE infections limit treatment options and are associated with high mortality rates, particularly among critically ill and immunocompromised patients (Table 1).

The study also identified several risk factors associated with CRE infections in the hospital setting. The results showed that patients with bedsores had a significantly higher proportion of CRE (34%) than those without (13.8%, p=0.034); bedsores appear to contribute to CRE infections, possibly due to the increased risk of skin and soft tissue breakdown in these patients. In addition, older patients were more likely to demonstrate CRE infections (34.5%) than younger patients (17.24%, p=0.069), indicating that the elderly population may be more susceptible to these problematic MDR pathogens. Importantly, this between-group difference was not statistically significant.

The high prevalence (>50%) of CRE among *K. pneumoniae* isolates is consistent with concerning trends reported by other studies out of Saudi Arabia. One study found that 36.1% of *K. pneumoniae* isolates from blood cultures in King Fahd Medical City in Riyadh were CRE [22]. Similarly, another study reported a significant increase in carbapenem resistance rates, from 6.6% for imipenem in 2011 to 59.9% in 2021, in a tertiary care hospital in Makkah, Saudi Arabia [23]. These findings highlight the critical level of antibiotic resistance among *K. pneumoniae* infections in healthcare facilities across the region.

Importantly, we found a strong association between pressure ulcers and the development of CRE infections (p=0.034). Previous studies [19, 20] have reported high rates of antibiotic resistance, particularly to carbapenems, among *K. pneumoniae* isolates in healthcare facilities in Saudi Arabia. These studies emphasized the importance of addressing underlying risk factors, such as preventing and managing pressure ulcers, to limit the spread of MDR pathogens [20,21].

The specific association between age and CRE infections is also consistent with another recent study where the findings indicated that adults were at higher risk of* K. pneumoniae* BSIs than pediatric patients [22]. The study aimed to understand the prevalence of BSIs, their antimicrobial resistance, and their impact on patients. The researchers identified 152 cases of BSIs caused by *K. pneumoniae* and found adults (66.4%) to be more susceptible than children (33.6%); they also observed slightly more infections in females than males. Notably, neurological disorders emerged as the leading underlying condition for BSIs across all ages, with the highest mortality rates observed among adults with multi-organ dysfunction. The infections resisted numerous antibiotics, including amoxicillin-clavulanate, cefuroxime, ceftazidime, cephalothin, and carbapenems. The study concluded that *K. pneumoniae *BSIs affect the community and are predominantly associated with MDR infections [22].

Thus, the elderly in long-term care facilities may be more susceptible to MDR infections, underscoring the need for targeted interventions and enhanced surveillance in this patient group [22]. The ubiquitous use of Foley catheters among the current study’s patients, in the absence of a significant association with resistance patterns, is consistent with the results of a prior study. *K. pneumoniae* was determined to be a major cause of intensive care unit infections and carries a high mortality rate. Still, the use of invasive devices was not the primary driver of the observed MDR.

This study reveals a high prevalence of CRE and ESBL-producing *K. pneumoniae* in a long-term care hospital in Riyadh, Saudi Arabia. The results highlight an urgent need to strengthen infection control measures, particularly among male patients in this environment.

Our results should be considered alongside several specific study limitations: the hospital had a limited bed rotation rate and bed capacity, resulting in a relatively small sample size. Moreover, no female subjects were included as the female ward was under renovation during the study period.

## Conclusions

In conclusion, the results of this study highlight the concerning prevalence of MDR *K. pneumoniae*, particularly CRE, in long-term healthcare hospitals. This study underscores the risk factors of pressure ulcers, advancing age, and the high rates of multidrug resistance, emphasizing the urgent need for strategies that comprehensively address these challenging healthcare-associated infections. Conducting an epidemiological surveillance study on* K. pneumoniae* in long-term care hospitals on female patients is recommended. Such research may improve our understanding of this infection and enhance our knowledge of antibiotic management.
